# Bis(5-chloroquinolin-8-olato)-bis(pyridine)-cobalt(II) as new catalytic material

**DOI:** 10.1038/s41598-022-06312-6

**Published:** 2022-02-09

**Authors:** Joanna Drzeżdżon, Celina Mokwa, Artur Sikorski, Patrycja Parnicka, Adriana Zaleska-Medynska, Jacek Malinowski, Magdalena Kwiatkowska, Barbara Gawdzik, Dagmara Jacewicz

**Affiliations:** 1 Faculty of Chemistry, University of Gdańsk, Wita Stwosza 63, 80-308 Gdańsk, Poland; 2 Institute of Chemistry, Jan Kochanowski University, Świętokrzyska 15 G, 25-406 Kielce, Poland

**Keywords:** Chemistry, Materials science

## Abstract

Nowadays, studies are carried out on the design and synthesis of new catalysts for olefin oligomerization and polymerization, which would contain non-toxic metals and at the same time show high catalytic activities. Complex compounds of transition metal ions such as Fe(II), Cr(III) and Zr(II) containing pyridine or quinoline as ligands show at least moderate catalytic activity in ethylene and propylene polymerizations. To investigate the catalytic activity of the complex containing pyridine ligands and quinoline derivatives, here we have synthesized the crystals of new bis(5-chloroquinolin-8-olato)-bis(pyridine)-cobalt(II) solvate. The synthesized cobalt(II) complex compound was tested in reactions of 2-chloro-2-propen-1-ol and norbornene oligomerizations. Our studies showed that bis(5-chloroquinolin-8-olato)-bis(pyridine)-cobalt(II) after activation by MMAO-12 catalyzes the formation of oligomers in nitrogen atmosphere, under atmospheric pressure and at room temperature. Bis(5-chloroquinolin-8-olato)-bis(pyridine)-cobalt(II) possesses moderate catalytic activity in the formation of norbornene oligomers process and low catalytic activity in 2-chloro-2-propen-1-ol oligomerization.

## Introduction

Polymer materials are produced in the global industry by the free radical polymerization method or with the use of a catalyst containing a metal atom or cation^1,2^. Organometallic complexes are known as olefin oligomerization and polymerization catalysts^3^. In industrial olefin oligomerization and polymerization, the Ziegler–Natta catalysts were used firstly, but despite high catalytic activities, they required the use of large amounts of solvents for purification of oligomerization and polymerization products^4^. The new generation of catalysts replacing Ziegler–Natta catalysts was metallocene compounds. They were metallocene complex compounds of transition metal atoms or cations, usually containing cyclopentadienyl and its derivatives as ligands^5^. Metallocene catalysts had some flaws, such as decomposition of the catalyst after activation by methylaluminoxane or an organoaluminum compound used as activator. Additionally, the oligomerization and polymerization reactions had to be carried out at high temperatures and high pressures^6^. The search for more sustainable catalysts than metallocene compounds resulted in the discovery of post-metallocene catalysts. These are complex compounds of transition metal ions, which catalyze oligomerization and polymerization reactions under low-pressure conditions^6^. Recent developments in post-metallocene catalysts have led to explore the complex compounds of transition metal ions such as Fe(II), Cr(III) and Zr(II) containing pyridine or quinoline as ligands, showing at least moderate catalytic activity in ethylene and propylene polymerizations^7–9^.

Among others, bis(5-chloroquinolin-8-olato)-bis(pyridine)-cobalt(II) solvate hypothetically could be applied as catalysts in polymerization processes, due to cobalt(II) complexes are known as olefin polymerization catalysts. The crystal structure of bis(5-chloroquinolin-8-olato)-bis(pyridine)-cobalt(II) ethanol solvate was described by Zhang et al. (CSD REFCODE: IVUMIX) in 2016 and his anticancer activity has been proved^10^. However, neglected area in the field of this compound is preparation in the presence of other solvents as well as studies on the catalytic activity of this complex compound.

Thus, we have synthesized a new polymorphic form, namely the bis(5-chloroquinolin-8-olato)-bis(pyridine)-cobalt water solvate (1:2) by changing synthesis and crystallization route, and tested it in the oligomerization reaction of 2-chloro-2-propen-1-ol. The products of oligomerization were characterized by the following methods: MALDI-TOF-MS, TG and SEM. The catalytic activity of bis(5-chloroquinolin-8-olato)-bis(pyridine)-cobalt(II) has been determined.

## Results and discussion

**Figure 1:**
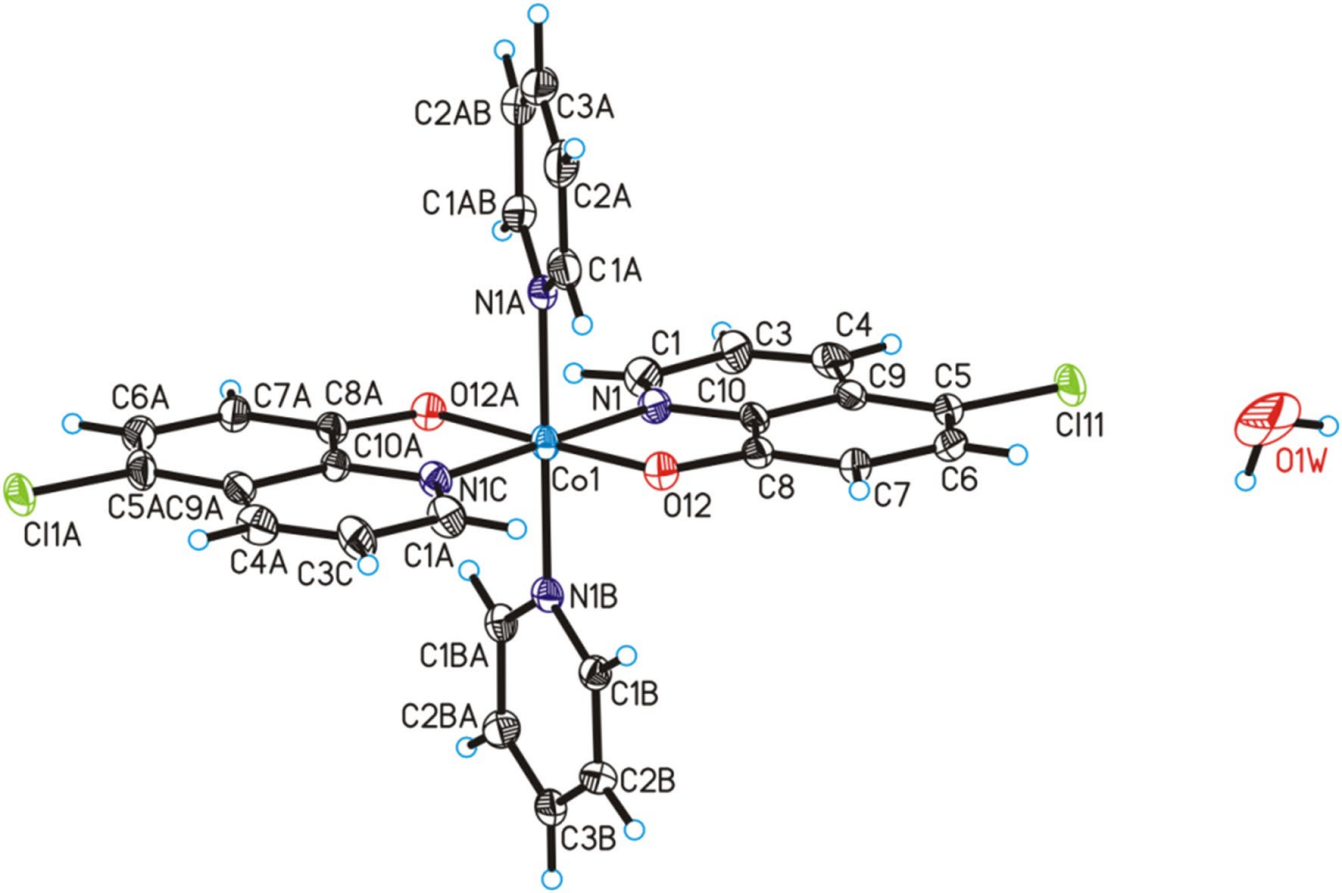
Molecular structure of the title compound in the crystal showing the atom labelling scheme and 25% probability displacement ellipsoids (H atoms are spheres of arbitrary size; disordered part of water molecule have been omitted).

**Figure 2:**
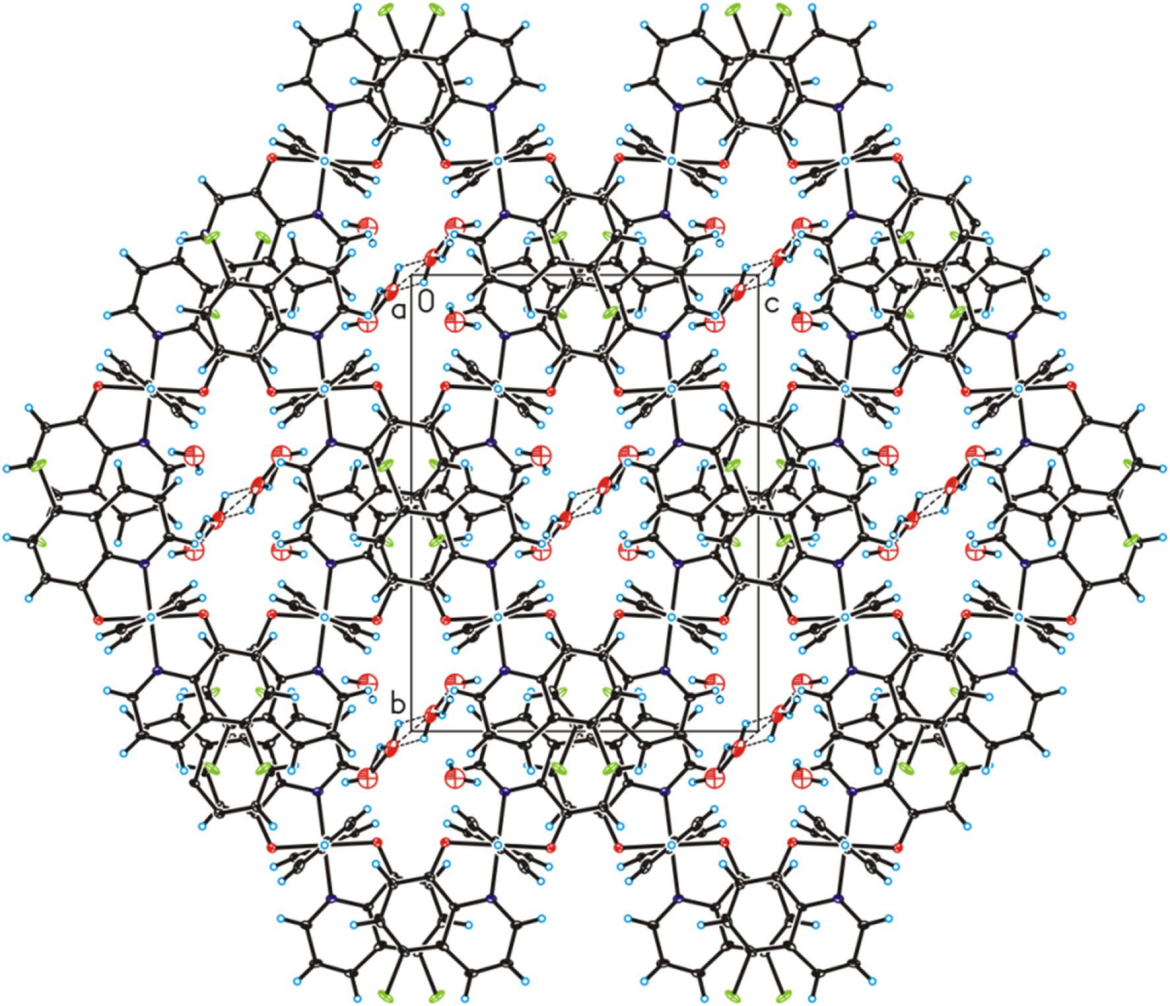
Crystal packing of title compound viewed along the *a*-axis (hydrogen bonds are represented by dashed lines).

The complex compound bis(5-chloroquinolin-8-olato)-bis(pyridine)-cobalt is known in the literature^10^, but its polymorphic form has been synthesized by our group and its physicochemical and catalytic properties have been described in this report. The bis(5-chloroquinolin-8-olato)-bis(pyridine)-cobalt water solvate (1:2) has been structurally characterized using single-crystal XRD method. The molecular structure of title compound has been shown in Fig. 1. The crystallographic data for title compound have been collected in Table 1. Title compound crystallize in the orthorhombic *P*nna space group with half bis(5-chloroquinolin-8-olato)-bis(pyridine)-cobalt(II) molecule and one disordered water molecule in asymmetric unit (Fig. 1) and is isostructural with bis(5-bromoquinolin-8-olato)-bis(pyridine)-cobalt(II) water solvate (1:2) (CSD REFCODE: EKISUO)^11^. In the crystal structure of the title compound, the geometric parameters (bond lengths and angles) characterized structure of cobalt(II) complex are similar to those observed in the crystal structure of EKISUO (Fig. 1). The Co(1) atom is six-coordinated in an octahedral coordination geometry, in which two 5-chloro-8-hydroxyquinolinium and two pyridine ligands. In the crystals, neighboring molecules of cobalt(II) complex are linked via weak C–H⋯π and π⋯π interactions to form porous metal–organic framework in voids of which disordered solvent molecules are located (Fig. 2). The polymorph obtained by our group of the compound crystallizes in triclinic crystal system and has a molecular weight of 574.32 g mol^−1^^10^, immediately the compound described in this study in orthorhombic crystal system and has a molar mass of 610.34 g mol^−1^. Whereas bis(5-bromoquinolin-8-olato)-bis(pyridine)-cobalt(II) water solvate (1:2) crystallizes in the orthorhombic crystal system and has the *P*nna space group as well as the compound studied by our group. The cobalt(II) complex with pyridine and 5-chloro-8-hydroxyquinoline described by Zhang et al.^10^ is a polymorph of the complex described in our report. It is the same chemical substance—one cobalt(II) cation bonded with two pyridine molecules and two 5-chloro-8-hydroxyquinoline anions but this complex occurs as different crystallographic form. These two polymorphs differ in unit cell dimensions (a, b, c) and crystallize in a different crystal system. Additionally, the complex described in our report has two crystallization water molecules. Therefore, the compound reported by Zhang et al.^10^ has a different molar mass than that described in this work.

**Table 1:** Crystallographic data for title compound.

Chemical formula	C_28_H_24_Cl_2_CoN_4_O_4_
FW/g mol^−1^	610.34
*T*/K	295(2)
λ_Mo_	0.71073 Å
Crystal system	orthorhombic
Space group	*P*nna
*a*/Å	14.4354(9)
*b*/Å	16.2400(9)
*c*/Å	12.3907(9)
*V*/Å^3^	2904.8(3)
*Z*	4
*ρ*_*cal*c_/g cm^−3^	1.396
*F(000)*	1252
*µ*/mm^−1^	0.814
2*θ* range for data collection/º	3.315–25.00
Completeness *2θ* /%	98.9
Reflections collected	19,092
Reflections unique	2541 [R_(int)_ = 0.1097]
Data/restraints/parameters	2541 / 36 / 229
Goodness-of-fit on *F*^2^	1.010
Final R_1_ value (*I* > 2*σ* (*I*))	0.0734
Final *w*R_2_ value (*I* > 2*σ* (*I*))	0.1877
Final R_1_ value (all data)	0.1490
Final *w*R_2_ value (all data)	0.2417
CCDC number	2,067,841

Elemental analysis of synthesized bis(5-chloroquinolin-8-olato)-bis(pyridine)-cobalt(II) confirmed the composition of the complex compound: C (55.12%), H (3.87%), N (17.93%). Analytically calculated: C (55.05%), H (3.93%), N (18.04%). The structure of bis(5-chloroquinolin-8-olato)-bis(pyridine)-cobalt(II) has been confirmed by spectroscopic methods such as IR, NMR and MALDI-TOF-MS (Supplementary Information). The IR spectrum shows that peak at 3064.07 cm^−1^ confirms the presence of C_(arom.)_–H vibrations. The presence of C_(arom.)_–O and C_(arom.)_=N has been identified by peaks at 1084.37 cm^−1^ and 1634.42 cm^−1^, respectively. The peaks at range 1447.10–1497.13 cm^−1^ confirms C=C_(aromatics)_ vibrations. The sharp and intense peak at 1040.93 cm^−1^ indicates the presence of vibrations from C_(arom.)_ to Cl. The stretching vibrations of Co–N and Co–O were identified by the presence of peaks at 555.18 cm^−1^ and 484.63 cm^−1^. The results of the IR tests are similar to the results obtained for another polymorphic form of the complex compound which are as follows^10^: “1573 (s, *ν*(C=N)), 1457 (s, *ν*(C–C)), 1084 (s, *ν*(C–O)), 1044 (s, *ν*(C–Cl)), 556 (s, *ν*(Co–N)), 486 (s, *ν*(Co–O))”.

MALDI-TOF-MS spectrum allow to indicate the [bis(5-chloroquinolin-8-olato)-bis(pyridine)-cobalt(II) − 2H_2_O + H]^+^ as peak at 576.89 m/z. The polymorphic form of bis(5-chloroquinolin-8-olato)-bis(pyridine)-cobalt(II) was tested by Zhang et al.^10^ using the ESI-MS method, which is a method similar to MALDI-TOF-MS. The results showed that polymorph fragmenting into the ion containing two DMSO molecules that was used as a solvent: [Co(5-chloroquinolin-8-olato)_2_ + 2DMSO + H]^+^^10^.

The ^1^H NMR results shows that peaks at 9.66 ppm identify the presence of protons N–C_(arom.)_–**H**, at 7.30 ppm O–C_(arom.)_–**H**, at 7.52 ppm Cl–C=C_(arom.)_–**H**. These results are compatible with the corresponding fragments of the isostructured compound to the title complex. They are as follows^11^: δ 9.69 ppm (N–C_(arom.)_–**H)**, 7.40 ppm (O–C_(arom.)_–**H)** and 7.69 ppm (Br–C=C_(arom.)_–**H**). The analysis of ^13^C NMR confirms the presence **C**–N–**C**_(arom.)_ (150.53 ppm) and other C atoms in pyridines rings (136.36 ppm, 126.55 ppm). Moreover the peaks at 164.21 ppm identify the presence of C atom connected by a binding to an O atom. The polymorph and the isostructural compound with bis(5-chloroquinolin-8-olato)-bis(pyridine)-cobalt(II) were not investigated by ^13^C NMR. When analyzing the literature data concerning the ^13^C NMR studies of ligands, i.e. pyridine and 5-chloro-8-hydroxyquinoline, it can be concluded that the ^13^C NMR results for the compound are correct, because peaks caused by the presence of different C atoms occur in the range 123.75–149.94 ppm and 111.47–152.95 ppm^12^ for pyridine and 5-chloro-8-hydroxyquinoline, respectively.

**Figure 3:**
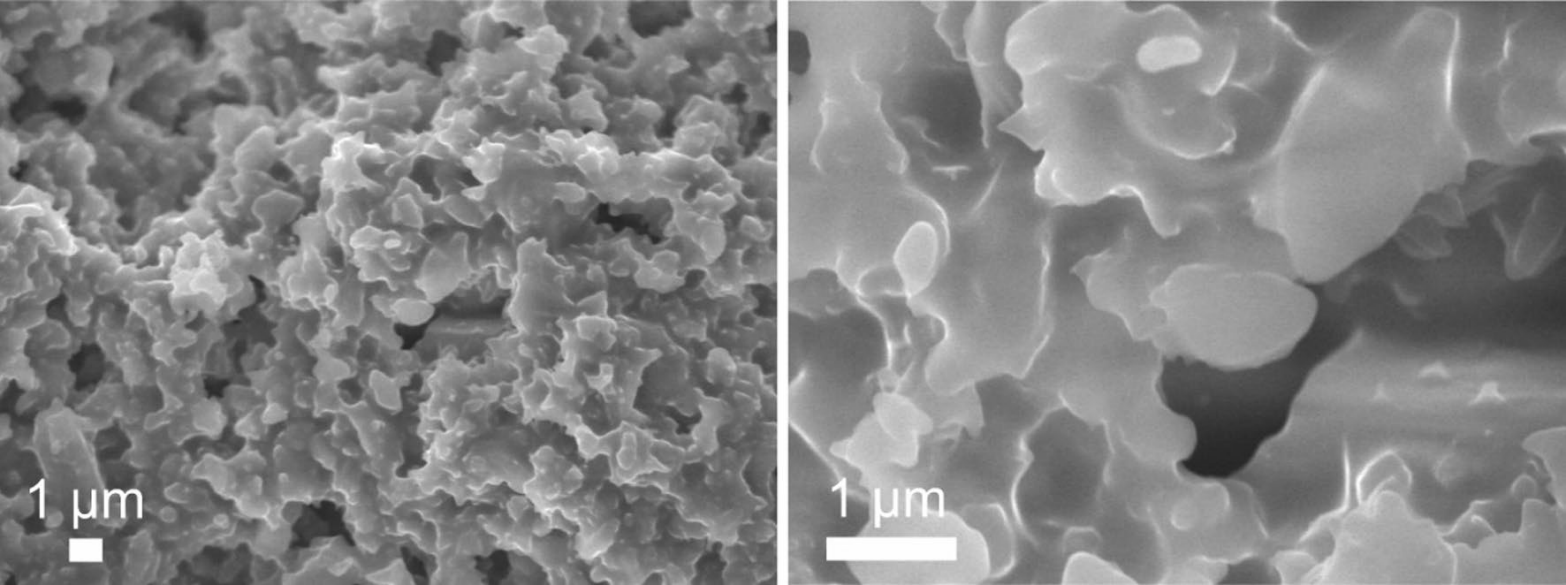
SEM images of bis(5-chloroquinolin-8-olato)-bis(pyridine)-cobalt(II) complex.

The porous structure of the bis(5-chloroquinolin-8-olato)-bis(pyridine)-cobalt(II) can clearly be seen from its scanning electron micrograph (SEM) as shown in Fig. 3. As observed, the complex is composed of aggregated flake-like particles with a size of several tens of micrometers. Moreover, the sample possesses not only macropores but also mesopores with a considerable volume. The hierarchically porous structure of the complex could be advantageous for a higher efficiency of the catalytic reactions. SEM studies of other cobalt(II) complex compounds, for example cobalt(II) complex compound with triphenylphosphine ligands, show that this material exists in micrometer-sized grains similar to the complex compound described in this paper^13^.

For the first time, the catalytic properties of bis(5-chloroquinolin-8-olato)-bis(pyridine)-cobalt(II) have been studies for 2-chloro-2-propen-1-ol and norbornene oligomerizations. Bis(5-chloroquinolin-8-olato)-bis(pyridine) -cobalt(II) as a new catalyst is characterized by the following features: it is a coordinating compound where the center of coordination is a cobalt(II) cation with a coordination number equal to 6, the catalyst has a molar mass equal to 610.34 g mol^−1^, the morphological structure of the new catalyst is flakes several dozen micrometers in size, it is well soluble in an equivolume mixture (1: 1) of DMSO and toluene, it must be activated by MMAO-12 for oligomerization of 2-chloro-2-propen-1-ol and norbornene, and after the reaction it cannot be reused due to the structure change by reaction with MMAO-12. The analysis of the MALDI-TOF-MS spectra showed that the oligomerization reaction of 2-chloro-2-propen-1-ol formed oligomers with 3–5 mers in the chain. The oligomerization of 2-chloro-2-propen-1-ol usually proceeds in such a way that a sample is produced containing oligomer chains of 5–15 mers^14^. In contrast, in the case of the norbornene oligomerization reaction, the product chains contain from 3 to 9 mers.

**Figure 4:**
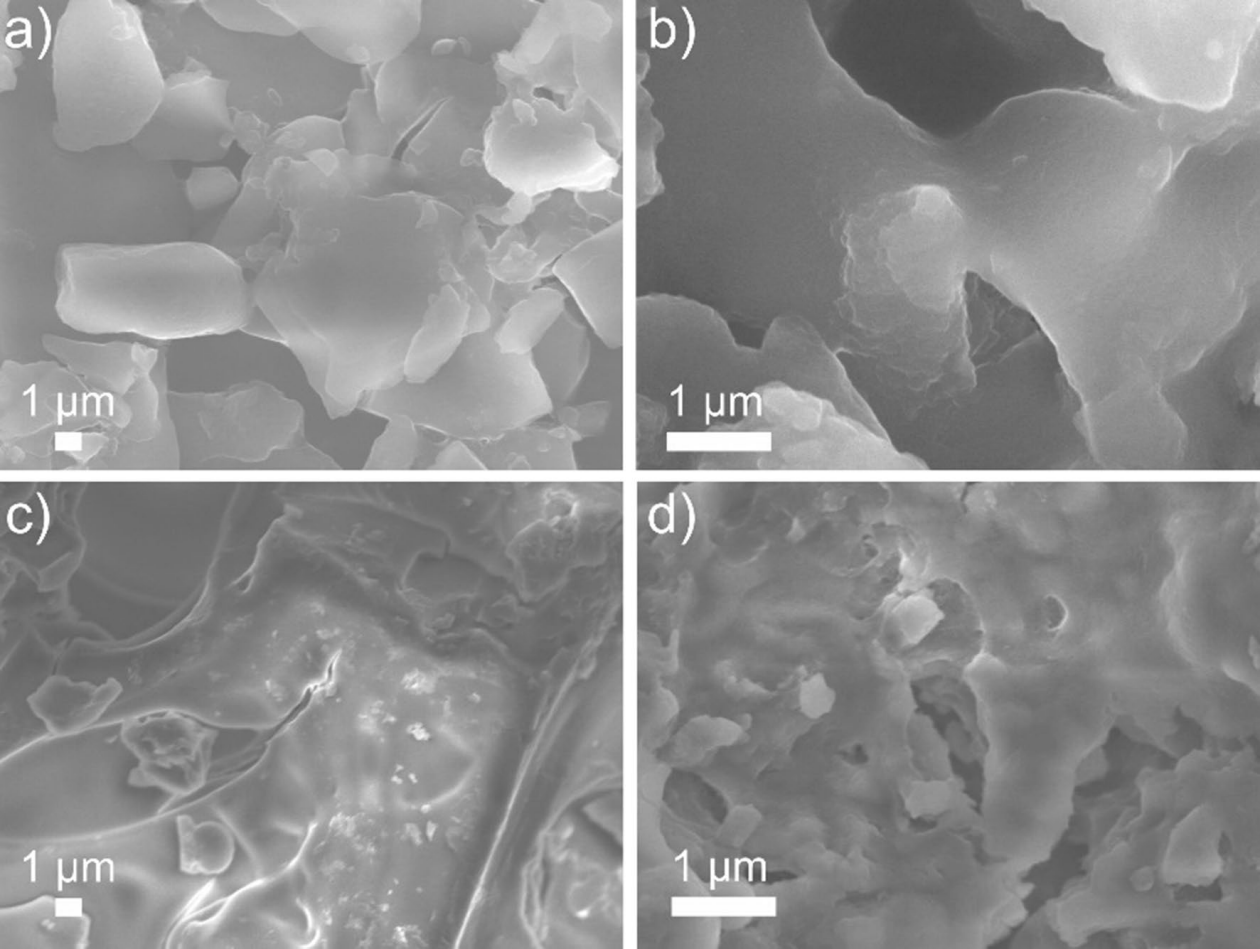
SEM images of (**a**, **b**) the products of 2-chloro-2-propen-1-ol oligomerization and (**c**, **d**) the products of norbornene oligomerization.

Thermal analysis of the oligomerization products allowed to study their thermal stability in the range from 20 to 1000 °C. The products of 2-chloro-2-propen-1-ol oligomerization thermally decompose within 4 steps while at 838.4 °C the product mass does not degrade further. During thermal decomposition, molecules of CO, CO_2_, HCl and H_2_O are released. In the case of the norbornene oligomerization reaction, the oligomers thermally decompose within 3 steps to temperature 947.9 °C where the product mass does not degrade further when increasing the temperature. At 280 °C the norbornene oligomers lose almost half of their mass, i.e. 43.6% of their mass. The morphological characterization of the products of 2-chloro-2-propen-1-ol oligomerization and the products of norbornene oligomerization are shown in Fig. 4. On the basis of the performed oligomerization reactions, the catalytic activity of the bis(5-chloroquinolin-8-olato)-bis(pyridine)-cobalt(II) complex was determined. For 2-chloro-2-propen-1-ol oligomerization the catalytic activity equal to 4.35 g mmol^−1^ h^−1^ bar^−1^. But for norbornene oligomerization the new precatalyst bis(5-chloroquinolin-8-olato)-bis(pyridine)-cobalt(II) complex exhibit the catalytic activity equal to 36.86 g mmol^−1^ h^−1^ bar^−1^. Taking into account the criterion of division of metallocene catalysts due to their catalytic efficiency introduced in 1999 by George J.P. Britovsek, Vernon C. Gibson and Duncan F. Wass, it can be concluded that bis(5-chloroquinolin-8-olato)-bis(pyridine)cobalt(II) when activated by MMAO-12 is low active catalyst for 2-chloro-2-propen-1-ol oligomerization and moderate active catalyst for the formation of norbornene oligomers process^15^. Noteworthy is the fact that the oligomerization reaction with the participation of the bis(5-chloroquinolin-8-olato)-bis(pyridine)cobalt(II) complex takes place in mild conditions (room temperature and atmospheric pressure).

Comparing the determined values of catalytic activity with other cobalt(II) complex compounds, it can be concluded that the new polymorph described in this work will produce relatively low rates of catalytic activity. Taking into account the general classification of catalysts in terms of their efficiency, it can be concluded that the new polymorph belongs to the catalysts with moderate or low activity^15^. Cobalt(II) complexes of bis(aryliminophosphoranyl)methane after activation by methylaluminoxane, exhibit moderately activity for ethylene polymerization at 20 °C^16^, while cobalt(II) complexes with bis(2,6-iminophosphoranyl)pyridine ligands after methylaluminoxane and triisobutylaluminum activation exhibit catalytic activity equal to 62 g mmol^−1^ h^−1^ bar^−1^^17^. During the polymerization reaction catalyzed by bis(2,6-iminophosphoranyl)pyridine cobalt(II) complexes, ethylene is introduced at a pressure of 10 bar at 25 °C^17^. Iminodiacetate cobalt(II) complex compound is highly active catalyst for 2-chloro-2-propen-1-ol oligomerization undergoing at normal pressure and at room temperature^14^. This complex compound exhibit catalytic activity equal to 759.04 g mmol^−1^ h^−1^ bar^−1^^14^. To our best knowledge, there are no cobalt(II) complex compounds described in the literature, which would show higher catalytic activity in olefin oligomerization or polymerization than iminodiacetate cobalt(II) complex compound. By analyzing the effect of the type of metal center in the post-metallocene catalyst i.e. Fe(II), Cr(III) and Zr(II) containing pyridine, lactones, proline derivatives or quinoline as ligands, on the catalytic activity, it can be concluded that the presence of the cobalt (II) cation in the structure of the complex compound lowers the catalytic activity in the olefin oligomerization reaction^6,18–20^.

## Methods

### Syntheses

**Figure 5:**
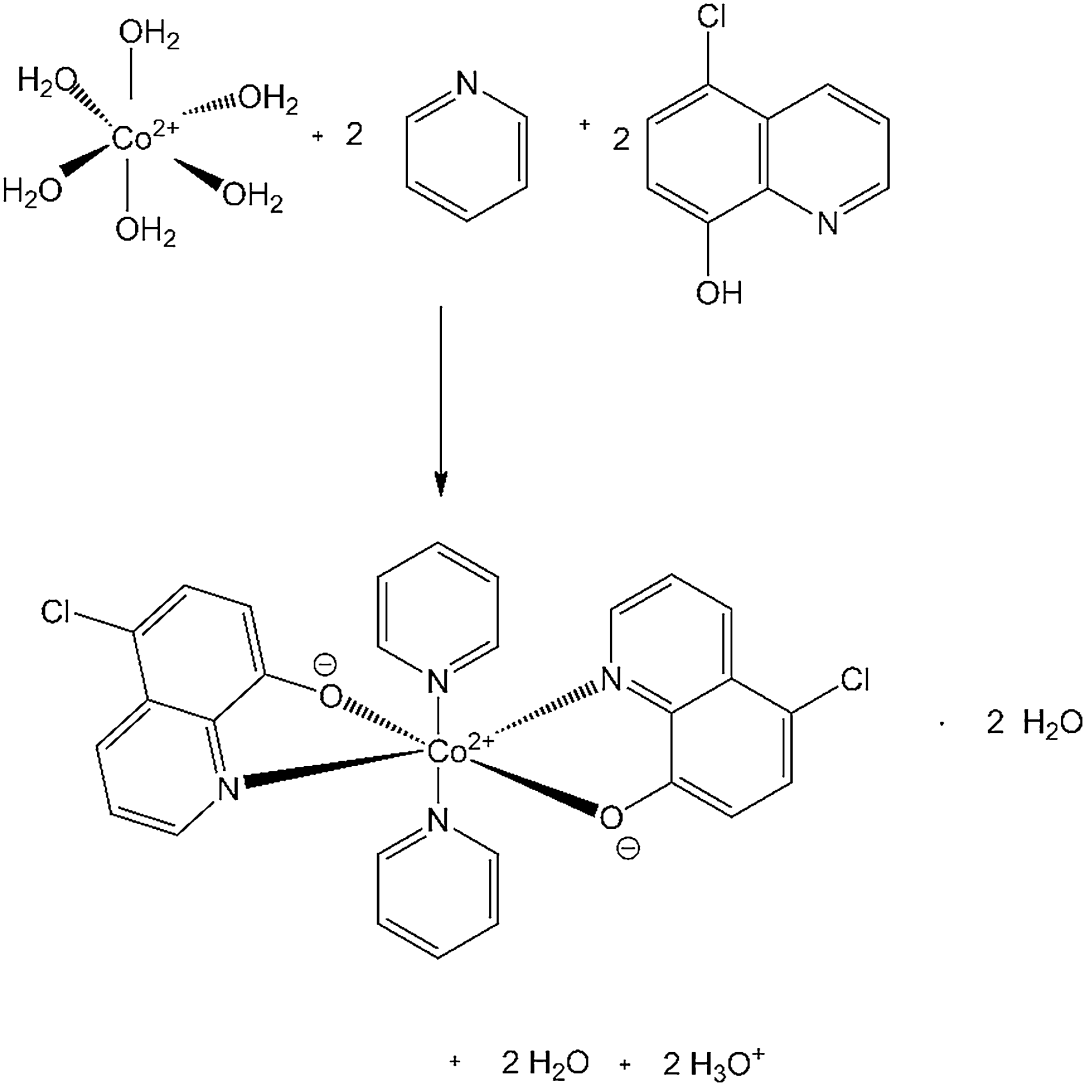
The diagram of formation of the bis(5-chloroquinolin-8-olato)-bis(pyridine)-cobalt(II) solvate.

The titled complex compound was synthesized by mixing 0.1 mmol (17.96 mg) of 5-chloro-8-hydroxyquinoline (purchased from Merck, 95 % purity) with 0.1 mmol (29.1 mg) Co(NO_3_)_2_·6 H_2_O (purchased from Acros Organics, 99 % purity) dissolved in a small amount of water. Then the mixture was poured with the solution prepared by mixing ethanol and pyridine in the 28: 1 volume ratio. The mixture has been heated at 80 °C in a round-bottomed flask until the color of the mixture changed. After cooling, brown crystals were obtained (Fig. 5).

**Figure 6:**
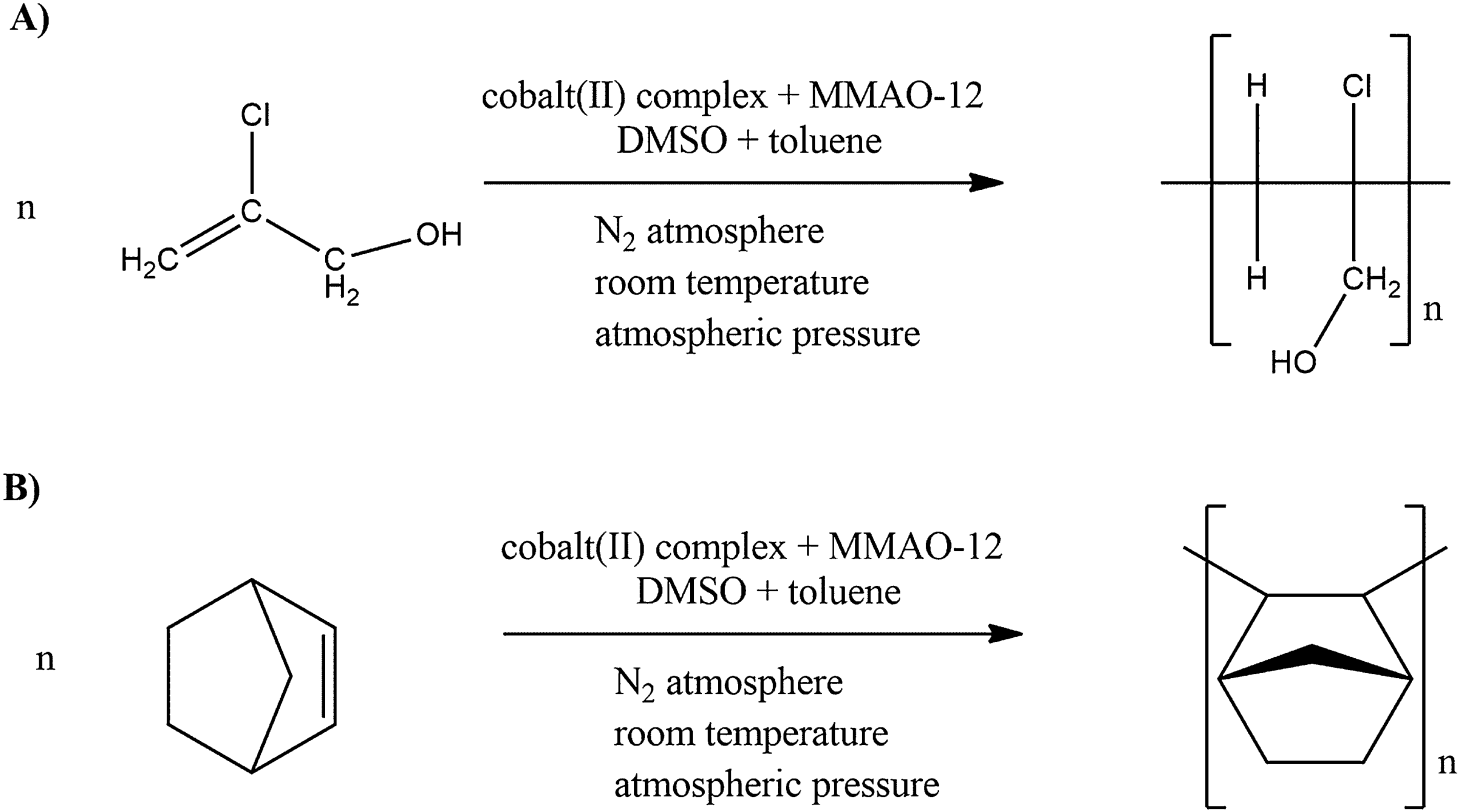
The scheme of oligomerization reaction (**A**) for 2-chloro-2-propen-1-ol and (**B**) for norbornene, where n denotes number of mers in the oligomer chain.

2-Chloro-2-propen-1-ol oligomerization (Fig. 6) was performed under a nitrogen atmosphere, under atmospheric pressure and at room temperature according to the following procedure: 3 µmol of the synthesized complex was placed in a glass cell, then dissolved in 1 mL of toluene and 1 mL of DMSO. Then, while stirring vigorously, 3 mL of MMAO-12 solution (MMAO-12, 7 wt% aluminum in toluene) was dropped into the cell. The molar ratio cobalt(II) compound:MMAO-12 was equal to 1:1000. The solution turned brown. In the next step, 1 mL of 2-chloro-2-propen-1-ol (purchased from Merck, 90 % purity) was dropped into the mixture of complex compound and MMAO-12. The molar ratio 2-chloro-2-propen-1-ol:cobalt(II) compound was equal to 37,667:1. The solution has been stirred over a magnetic stirrer until a gel was formed. Then the mixture was washed with hydrochloric acid 1M and methanol in 1:1 molar ratio.

Norbornene oligomerization (Fig. 6) was performed under a nitrogen atmosphere, under atmospheric pressure and at room temperature as follows: 3 µmol of the synthesized complex dissolved in the mixture 1 mL of toluene and 1 mL of DMSO. In the next step, 3 mL of MMAO-12 solution (MMAO-12, 7 wt% aluminum in toluene) was dropped into the cell. The molar ratio cobalt(II) compound:MMAO-12 was equal to 1:1000. As a result the solution turned brown. Next, 1.2 g of norbornene (purchased from Merck, 99 % purity) was dissolved in 1 mL of toluene. The molar ratio 2-chloro-2-propen-1-ol:cobalt(II) compound was equal to 4233:1. Then, 1 mL of the solution of norbornene was dropped into the cell. The solution has been stirred over a magnetic stirrer until a gel was formed. Then the mixture was washed with hydrochloric acid 1 M and methanol in 1:1 molar ratio.

### Physicochemical characteristics

Good-quality single-crystal of title compound was selected for the XRD measurements (295(2) K) and carried out on the Oxford Diffraction Gemini R ULTRA Ruby CCD diffractometer with the Mo *Kα* (λ = 0.71073 Å) radiation. CrysAlis has been used to determine the lattice parameters^21^. The structure of title compound was refined with the SHELXL program^22^. The geometrical calculations were made using the PLATON program^23^. The following programs PLUTO-78, ORTEPII and Mercury were used to an analysis and a presentation of molecular structures^24–26^.

Full crystallographic details of title compound have been deposited in the Cambridge Crystallographic Data Center (deposition No. CCDC 2067841) and they may be obtained from www: http://www.ccdc.cam.ac.uk, e-mail: deposit@ccdc.cam.ac.uk or The Director, CCDC, 12 Union Road, Cambridge, CB2 1EZ, UK.

Bis(5-chloroquinolin-8-olato)-bis(pyridine)-cobalt(II) has been examined with the elemental analysis using the Vario EL analyzer Cube (CHNS).

IR experiments were carried out in the range from 4000 to 600 cm^−1^ using a KBr pellet.

MALDI-TOF-MS spectra were recorded on a Bruker Biflex III spectrometer. 2,5-Dihydroxybenzoic acid (DHB) was served as a matrix.

The ^1^H and ^13^C NMR spectra were recorded using the Bruker Avance III 500 at 298 K. The complex compound was dissolved in deuterated-DMSO.

The thermogravimetric analysis of oligomers was performed using a TG209 Thermometer from Netzsch. The experiments were carried out in the temperature range from 20 to 1000 °C using the Al_2_O_3_ crucible.

The morphologies of the as-prepared samples were investigated using field-emission SEM (JSM-7610F, JEOL).

## Conclusions

We have synthesized the crystals of new bis(5-chloroquinolin-8-olato)-bis(pyridine)-cobalt(II) solvate. Bis(5-chloroquinolin-8-olato)-bis(pyridine)-cobalt(II) complex has been studied for the first time as the precatalyst for 2-chloro-2-propen-1-ol and norbornene oligomerizations. The bis(5-chloroquinolin-8-olato)-bis(pyridine)-cobalt(II) after activation by MMAO-12 was found to be an active catalyst for the formation of norbornene oligomers process. No significant activity was observed for 2-chloro-2-propen-1-ol oligomerization. The obtained products of the oligomerization reaction are thermally unstable at a temperature above 60 °C.

## Supplementary Information

Supplementary Figures.
