# Supplementary material for: Bis(5-chloroquinolin-8-olato)-bis(pyridine)-cobalt(II) as new catalytic material
Source: Sci Rep. 2022 Feb 9;12:2151. doi: 10.1038/s41598-022-06312-6 (PMC8828837; doi:10.1038/s41598-022-06312-6)
Supplement: Supplementary file 1 — Supplementary Figures. [file 41598_2022_6312_MOESM1_ESM.pdf]

Supplementary Information

Bis(5-chloroquinolin-8-olato)-bis(pyridine)-cobalt(II) as new catalytic material

Joanna Drzeżdżon [1], Celina Mokwa [1], Artur Sikorski [1], Patrycja Parnicka [1], Adriana Zaleska-Medynska [1], Jacek Malinowski [1], Magdalena Kwiatkowska [2], Barbara Gawdzik [2,*], Dagmara Jacewicz [1,*]

[1] Faculty of Chemistry, University of Gdańsk, Wita Stwosza 63, 80-308 Gdańsk, Poland; joanna.drzezdzon@ug.edu.pl (J.D.), celina.mokwa@wp.pl (C.M.), artur.sikorski@ug.edu.pl (A.S.), patrycja.parnicka@phdstud.ug.edu.pl (P.P.), adriana.zaleska-medynska@ug.edu.pl (A.Z.-M.), jacek.malinowski@phdstud.ug.edu.pl (J.M.), dagmara.jacewicz@ug.edu.pl (D.J.),

[2] Institute of Chemistry, Jan Kochanowski University, Świętokrzyska 15 G, 25-406 Kielce, Poland; magdalena.kwiatkowska@ujk.edu.pl (M.K.), b.gawdzik@ujk.edu.pl (B.G.)

* Correspondence: b.gawdzik@ujk.edu.pl (B.G.),dagmara.jacewicz@ug.edu.pl (D.J.)

Table of contents

1. **Structure of bis(5-chloroquinolin-8-olato)-bis(pyridine)-cobalt(II)……………….3**
2. **The studies of 2-chloro-2-propen-1-ol oligomerization……………………..........5**
3. **The studies of norbornene oligomerization ……………………………………...6**

1. Structure of bis(5-chloroquinolin-8-olato)-bis(pyridine)-cobalt(II)

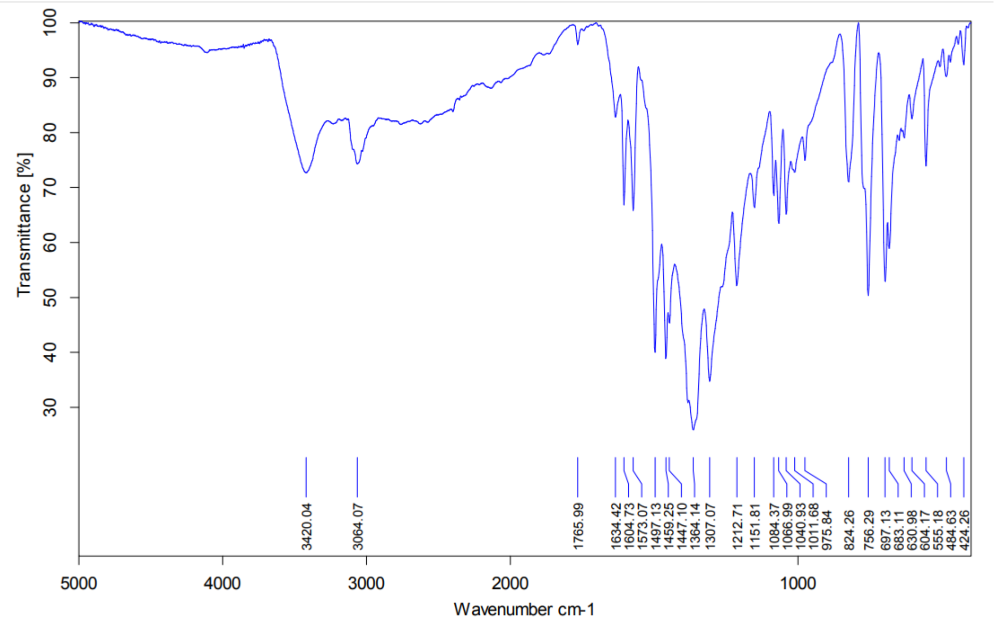

Figure 1. IR spectrum of bis(5-chloroquinolin-8-olato)-bis(pyridine)-cobalt(II).

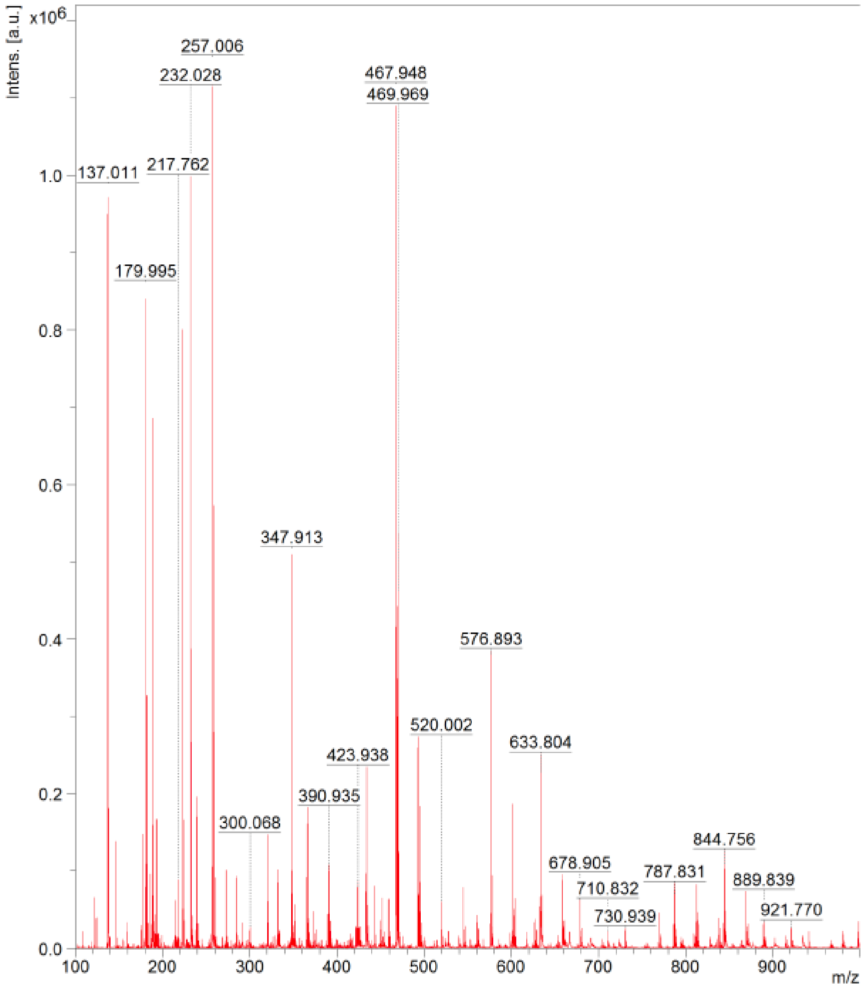

Figure 2. MALDI-TOF-MS spectrum of bis(5-chloroquinolin-8-olato)-bis(pyridine)-cobalt(II).

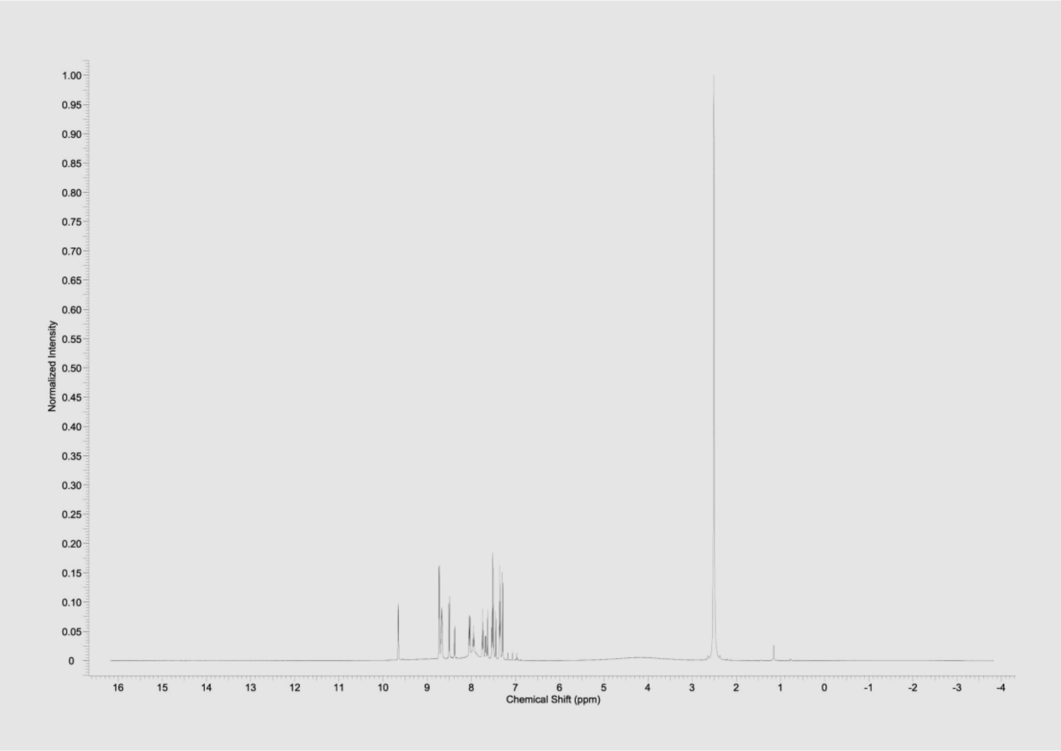

Figure 3. ^{1}H NMR spectrum of bis(5-chloroquinolin-8-olato)-bis(pyridine)-cobalt(II).

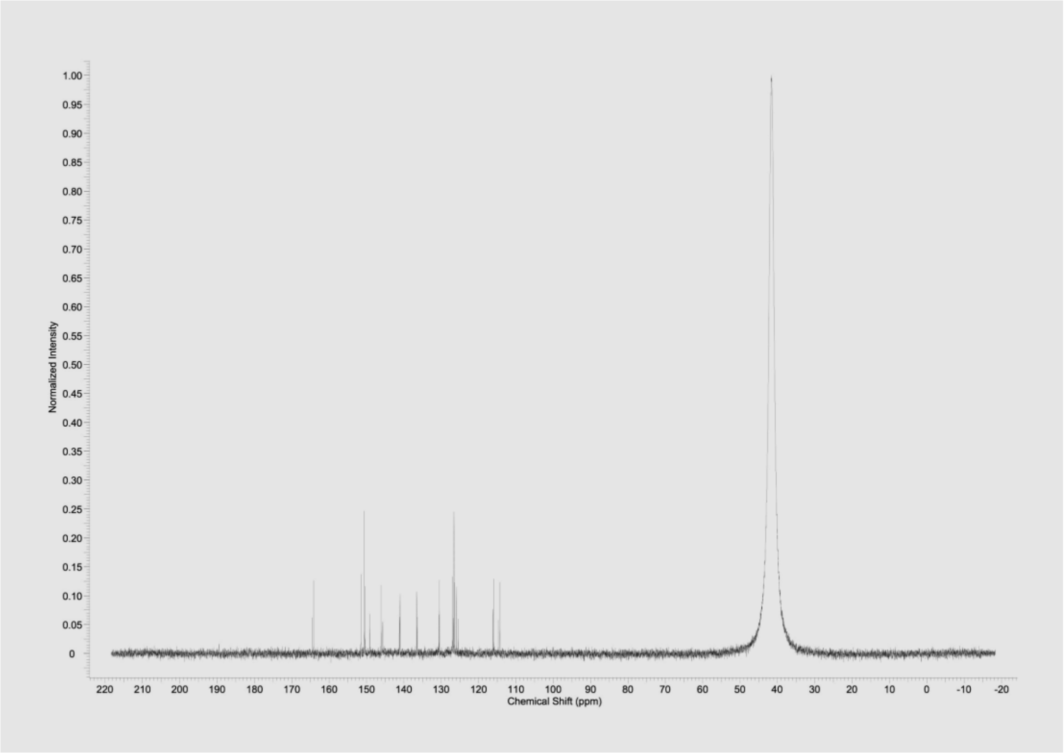

Figure 4. ^{13}C NMR spectrum of bis(5-chloroquinolin-8-olato)-bis(pyridine)-cobalt(II).

2. The studies of the 2-chloro-2-propen-1-ol oligomerization

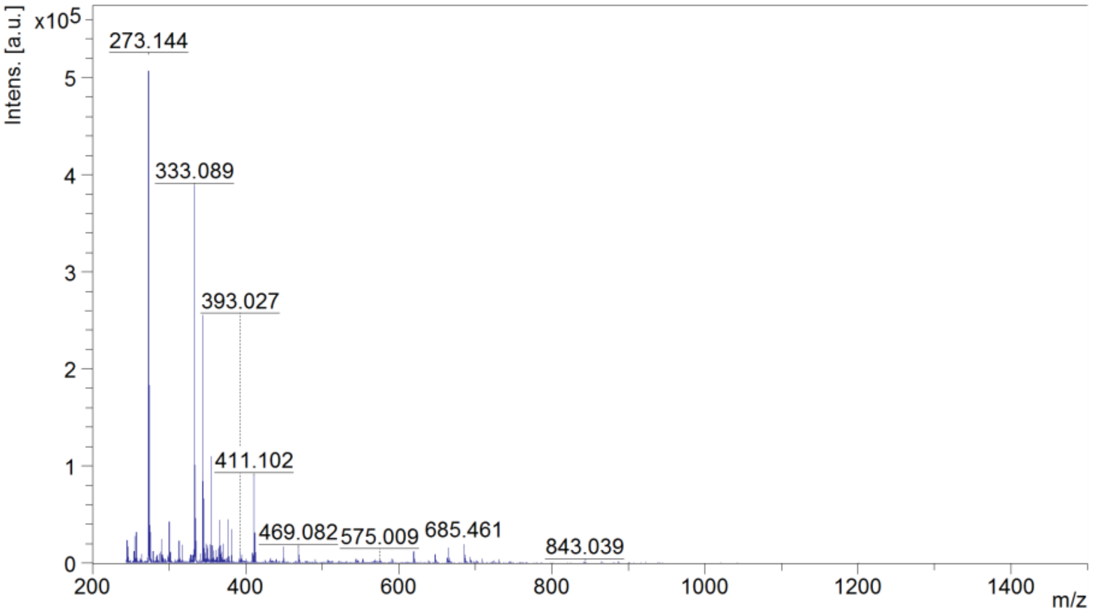

Figure 5. MALDI-TOF-MS spectrum of oligomers.

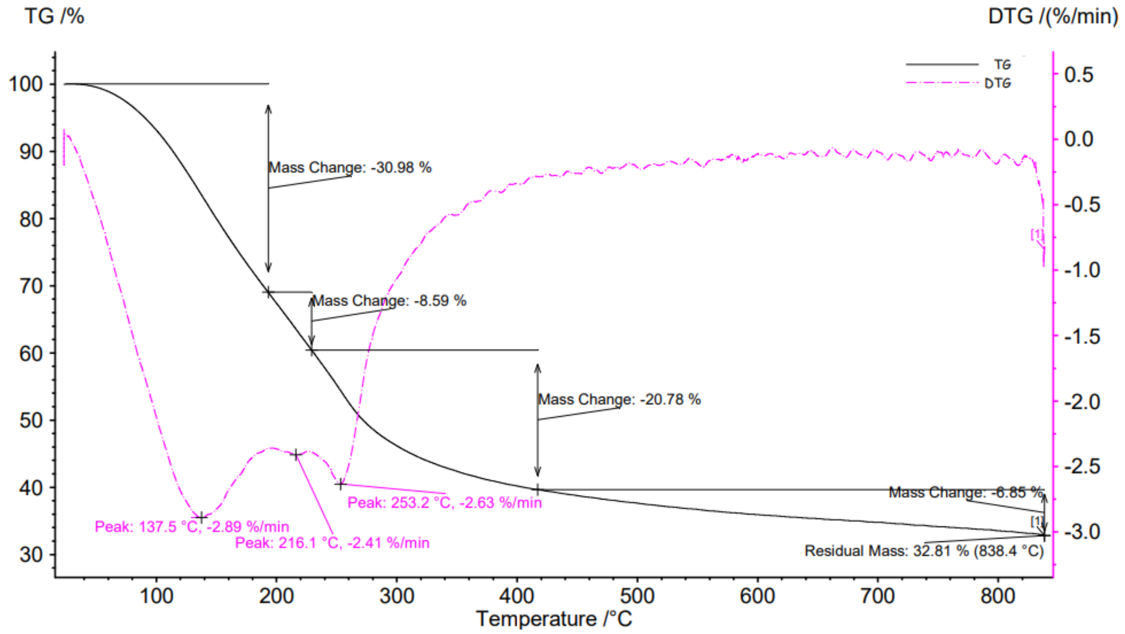

Figure 6. Results of TG analysis of oligomers.

3. The studies of the norbornene oligomerization

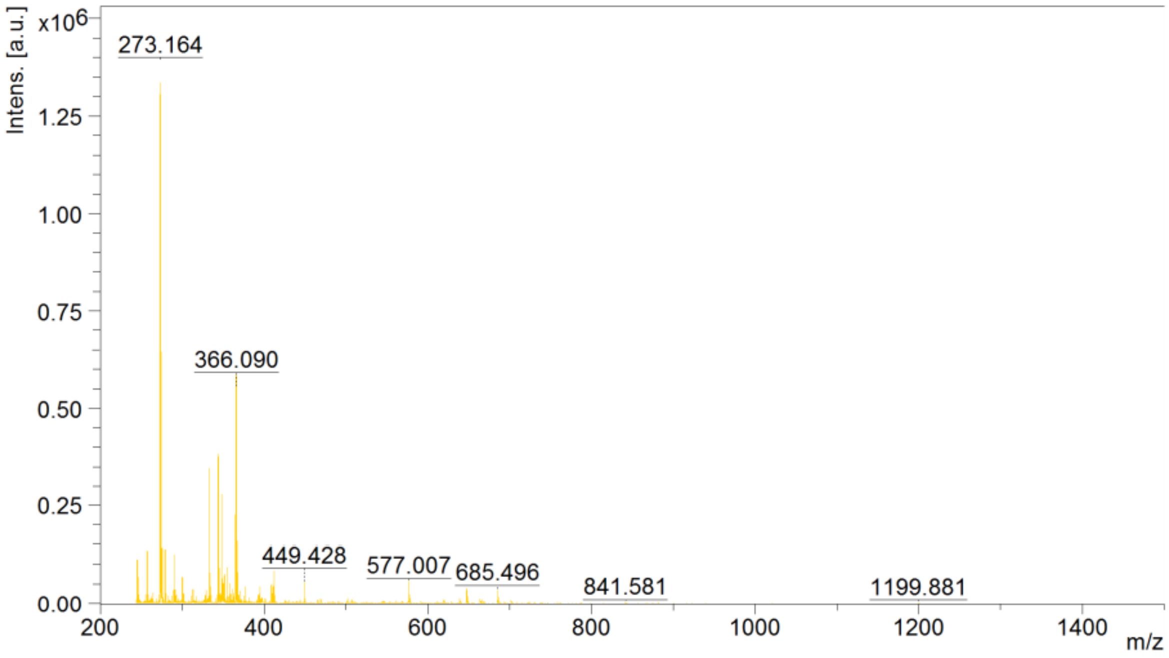

Figure 7. MALDI-TOF-MS spectrum of oligomers.

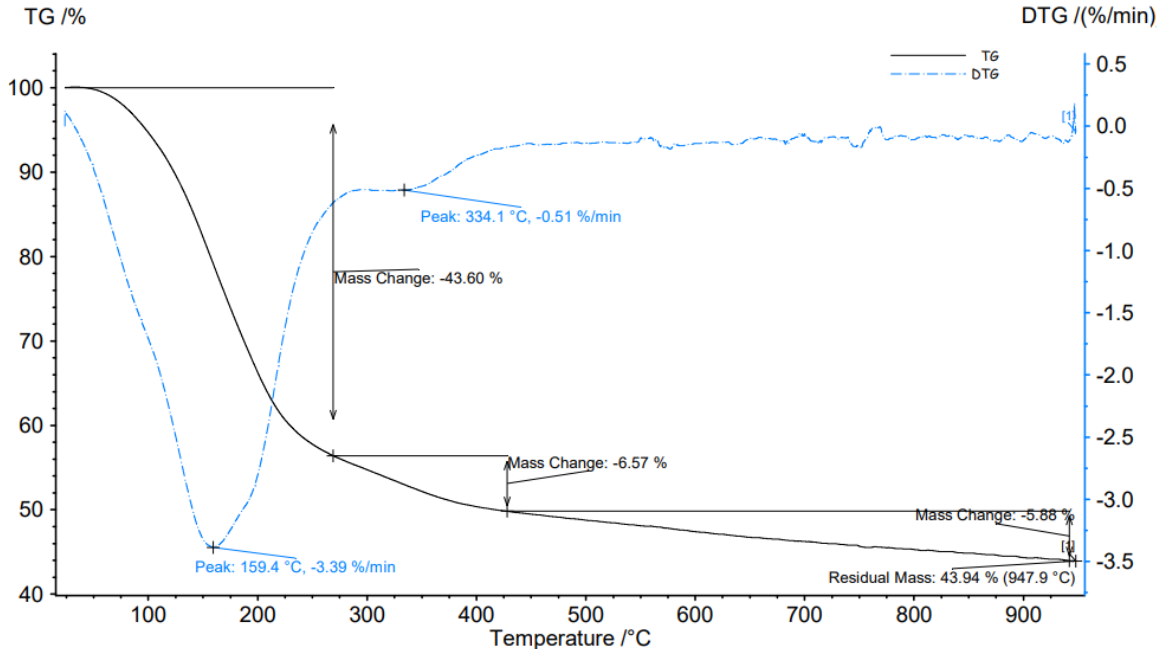

Figure 8. Results of TG analysis of oligomers.
